# The Mutation of Glu at Amino Acid 3838 of AtMDN1 Provokes Pleiotropic Developmental Phenotypes in Arabidopsis

**DOI:** 10.1038/srep36446

**Published:** 2016-11-08

**Authors:** Peng-Cheng Li, Shao-Wei Yu, Ke Li, Jin-Guang Huang, Xing-Jun Wang, Cheng-Chao Zheng

**Affiliations:** 1 State Key Laboratory of Crop Biology, College of Life Sciences, Shandong Agricultural University, Tai’an, Shandong, PR China; 2 Bio-Tech Research Center, Shandong Academy of Agricultural Sciences, Shandong Provincial Key Laboratory of Crop Genetic Improvement, Ecology and Physiology, Jinan, Shandong, PR China; 3 College of Life Sciences, Shandong University, Ji’nan, Shandong, PR China

## Abstract

MDN1/Rea1, as an AAA-type ATPase, is predicted to be the largest protein involved in pre-ribosome maturation in most organisms. However, its function in plant growth and development is poorly understood. Here, we characterized a novel Arabidopsis mutant, *dwarf* & *short root (dsr*) *1*, which shows pleiotropic developmental phenotypes, such as slow germination, short root, dwarf shoot, and reduced seed set under normal growth conditions. Using positional cloning, we revealed that the AtMDN1 function is impaired by a ‘glutamic acid’ to ‘lysine’ change at position 3838 of the amino acid sequence in *dsr1*. Multiple sequence alignment analysis revealed that the mutated Glu residue, which located in the linker domain of AtMDN1, is extremely conserved among organisms. AtMDN1 is expressed in various tissues, particularly in the shoot apex and root tip. Moreover, the results of transcript profile analyses showed that the dysfunction of AtMDN1 in *dsr1* impairs the expression of genes related to plant growth and development, which is tightly associated with the pleiotropic phenotypes of *dsr1*. Thus, we concluded that the Glu residue plays a vital role in maintaining AtMDN1 functions, which are essential for plant growth and development.

Ribosomes translate genetic information from messenger RNA to proteins in cells. In eukaryotic cells, ribosome biosynthesis is a complicated process involving ribosomal RNA (rRNA) particle assembly and maturation in the nucleolus and nucleoplasm prior to cytoplasmic export^1^,^2^,^3^,^4^. rRNA processing is energy consuming^5^,^6^,^7^. Many of the non-ribosomal factors involved in the assembly of pre-rRNA particles are putative GTPases and AAA-ATPases (ATPases associated with various cellular activities)^2^,^3^,^8^.

MDN1/Rea1 (ribosome export associated), a dynein-related AAA-ATPase and component of the pre-60S ribosome, is predicted to be the largest protein in eukaryotic cells^9^,^10^,^11^. In yeast, Rea1 was successively identified from the Nug1-purified particle and the temperature-sensitive mutant *rix1-1*^8^,^12^. The Nug1-purified particle is a pre-60S ribosome at the transition from the nucleolus to the nucleoplasm, while Rix1 is associated with the late pre-60S ribosome in the nucleoplasm^8^,^12^. In tandem affinity purification (TAP) of the Rix1 particle, Rea1 is readily detected^8^. In *rix1-1*, the export of ribosomal proteins from the nucleoplasm to the cytoplasm is severely delayed at restrictive temperature^8^. Furthermore, the depletion of Rea1 leads to defects in pre-rRNA processing^1^,^8^. These observations suggest that Rea1 plays roles in pre-ribosome maturation and export.

The pre-60S ribosome confers a ‘tadpole-like’ structure with the ‘tail’ region comprising Rea1^1^. The Rea1 protein possesses four key domains, a N-terminal ATPase containing six tandem AAA repeats, a large linker, a small D/E-rich domain, and a C-terminal MIDAS (metal ion-dependent adhesion site)^10^,^13^. The six AAA protomers constitute a ring-shaped structure, which attaches to the Rix1 pre-ribosome^1^,^2^,^13^. The tail structure protruding from the pre-ribosome is flexible and comprises the linker, D/E-rich domain, and MIDAS^2^. According to the results of *in vivo* and *in vitro* experiments, the MIDAS domain, located at the end of the tail, is essential for the removal of Ytm1 and Rsa4 (pre-60S ribosome factors) at different critical transition points by binding to the MIDAS-interacting domain (MIDO) of these proteins^1^,^9^. However, the linker domain function remains poorly understood.

To date, few studies have reported Rea1 functions in plant development. In Arabidopsis, the loss-of-function MDN1 mutation resulting from a T-DNA insertion is lethal^14^. Recently, Qi *et al*. reported that a maize mutant with decreased Rea1 function, dek*, produces small kernels^15^. Plant growth and development is severely delayed as a result of impaired ribosome biogenesis in dek*^15^. These results suggest that Rea1 is vital for plant viability. In the present study, we characterized a novel Arabidopsis mutant, *dsr1*, that exhibits pleiotropic developmental phenotypes under normal growth conditions. Using map-based cloning, we demonstrated that the *dsr1* phenotypes were elicited by a site-mutation of a conserved ‘Glu’ in the linker domain of AtMDN1.

## Results

### Phenotypes of the *dsr1* mutant

**Figure 1:**
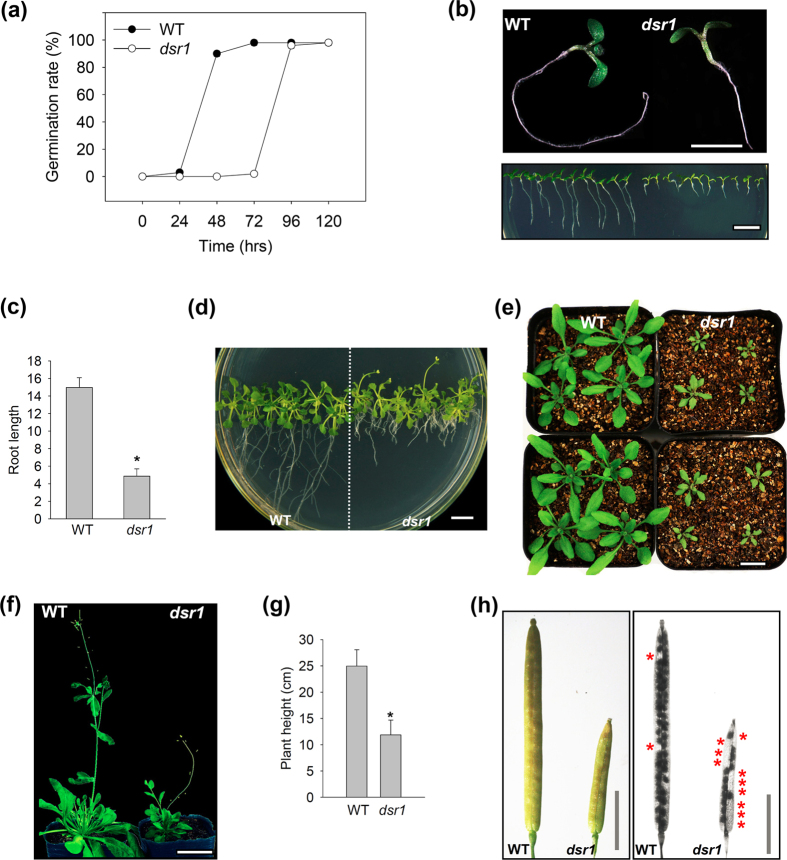
Phenotypes of *dsr1*. (**a**) Germination rate analysis of *dsr1* grown under normal growth conditions. (**b**) The 7-d-old seedling phenotypes of *dsr1*. Bar = 0.5 cm. (**c**) Root lengths of seedlings showed in (**b)**. (**d**) The 3-week-old plant phenotypes of *dsr1* grown under MS medium. The plants were initially grown on 1/2 MS medium for the first 7 days after germination, and subsequently transferred to new MS medium. Bar = 0.5 cm. (**e**) The 3-week-old plant phenotypes of *dsr1* grown in nutrient soil. The plants were initially grown on 1/2 MS medium for the first 7 days after germination, and subsequently transferred to nutrient soil. Bar = 1.0 cm. (**f**) The 5-week-old plant phenotypes of *dsr1* grown under normal growth conditions. (**g**) Plant height analysis of the 5-week-old *dsr1* and wild-type plants showed in (**f)**. (**h**) Silique and seed distribution phenotypes of wild type and *dsr1* selfed plants.

The *dsr1* mutant was screened from approximately 30,000 ethane methylsulfonate (EMS)-mutagenized Col-0 M2 seedlings. The *dsr1* mutant exhibits pleiotropic developmental phenotypes. As shown in Fig. 1a, the seed germination rate of *dsr1* was substantially lower than that of wild-type seedlings under normal growth conditions. The *dsr1* seedlings exhibited a severe short root phenotype (Fig. 1b). The root length of the mutant was less than half that of wild type (Fig. 1c). When plants were grown for 3 weeks, not only the roots but also the shoots of *dsr1* were severely stunted compared with wild type (Fig. 1d,e). Furthermore, at the reproductive growth stage, the plant height of *dsr1* was only half that of the wild type, indicating that *dsr1* possesses a dwarf phenotype (Fig. 1f,g). In addition, the siliques of *dsr1* were obviously short and produced approximately 50% of the seed set, while wild-type siliques were associated with nearly full seed set (Fig. 1h). Thus, in plant growth and development, the functions of some gene(s) might be disrupted in *dsr1*.

### Positional cloning of *dsr1*

**Figure 2:**
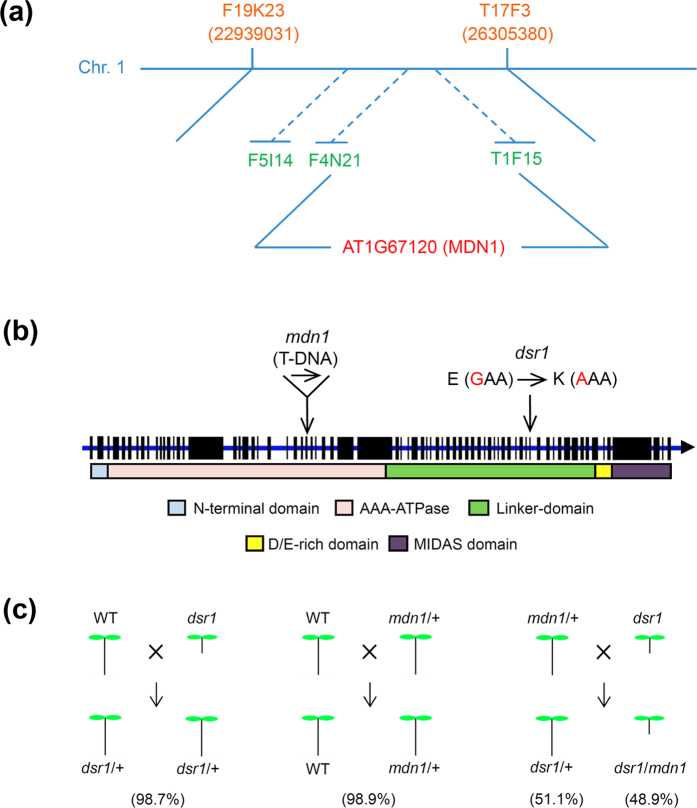
Map-based cloning of *dsr1*. (**a**) Map position of *dsr1* on chromosome 1 (*At1g67120*). Some of the BAC markers used in the present study are indicated. (**b**) The gene structure and protein domains of AtMDN1 are shown. The exons are shown as black boxes, and the introns are presented as blue lines. The *dsr1* mutation site and the inserted position of the T-DNA insertion *mdn1* mutant are indicated. (**c**) Complementary experiments of *dsr1*. The percentages of F1 plants with normal or short roots are indicated.

To isolate the target gene(s) responsible for the *dsr1* phenotypes, a positional cloning approach was applied. To remove the background mutations, the *dsr1* was backcrossed twice to wild-type (Col-0) plants. F1 plants were generated after crossing *dsr1* with Landsberg (an Arabidopsis wild ecotype), followed by self-fertilization for generation of the F2 population. The quantitative ratio of plants with normal phenotypes to the *dsr1* phenotypes in the F2 population was approximately 3:1, indicating that *dsr1* is a recessive mutant. Lots of BAC markers were used for map-based cloning, and the representatives are shown in Fig. 2a. F19K23 and T17F3 are coarse-mapping markers, and the other markers are fine-mapping markers (Fig. 2a). We mapped the target gene to *At1g67120 (MIDASIN1*/*AtMDN1*) and identified a G to A base substitution that causes an E to K substitution at position 3838 of the amino acid sequence (Fig. 2b). The mutation site of *dsr1* was located in the linker domain of AtMDN1 (Fig. 2b).

A T-DNA insertion mutant in the Col-0 ecotype of *AtMDN1*, SALK_057010 (*mdn1*), was obtained from the Arabidopsis Biological Resource Centre (ABRC, Fig. 2b). A previous study revealed that the *mdn1* homozygote is lethal, reflecting a disruption of the AAA-domain^14^. The *mdn1* heterozygote (*mdn1*/+) exhibits normal growth phenotypes (Supplementary Fig. 1). Reflecting the extremely long sequence of *AtMDN1* (5393 amino acids), it is hard to induce the full gene into *dsr1* or *mdn1*/+. Therefore, a genetic approach was performed for the complementary experiments. Reflecting the various phenotypes of *dsr1*, we employed the most intuitive phenotype, short root, as an indicator to analyse the segregation ratios of hybrid plants. As shown in Fig. 2c, most of WT × *dsr1* F1 seedlings (98.7%) developed normally under normal growth conditions, reflecting the fact that *dsr1* is a recessive mutant. In addition, similar results were observed in *mdn1*/+ × WT F1 plants (97.9%). However, approximately half of the *mdn1*/+ × *dsr1* F1 progeny seedlings exhibited significant short root phenotypes, reflecting the generation of *dsr1*/*mdn1* heterozygotes. These results further indicate that the *dsr1* phenotypes result from defects in AtMDN1 function.

### The mutated amino acid, Glu, is conserved in organisms

**Figure 3:**
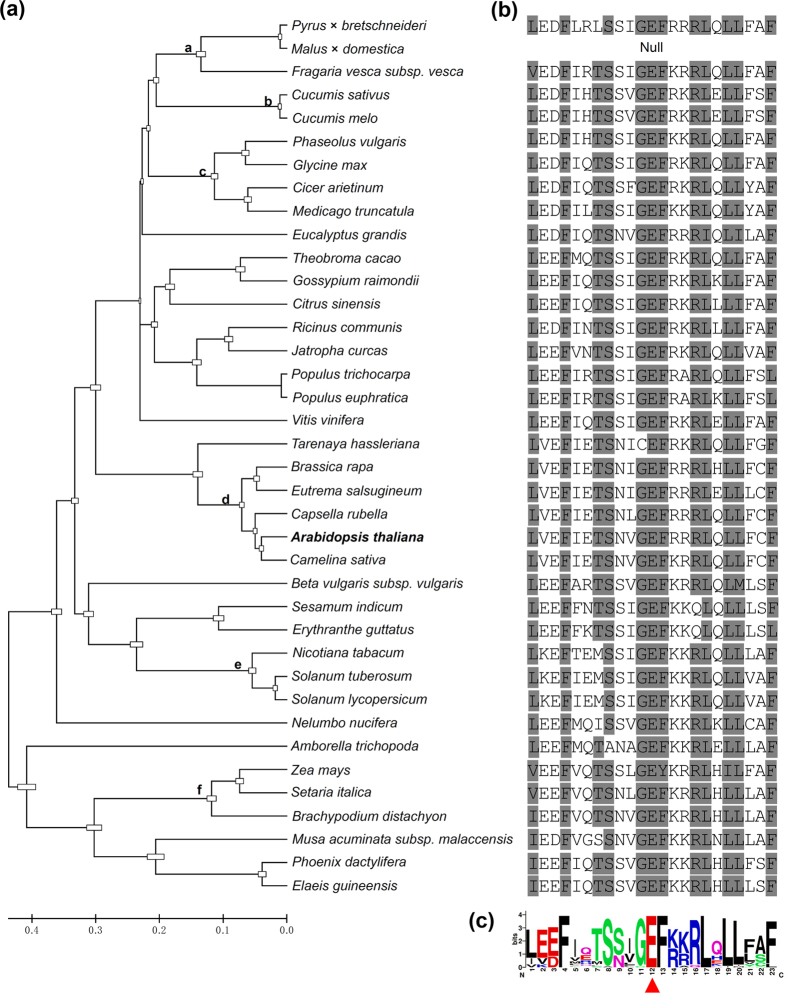
Amino acid sequence alignment of MDN1 homologues in plants. (**a**) Phylogenetic relationships of the MDN1 homologues in plants. The phylogenetic tree was constructed using MEGA 5. The lower case letters represent different plant categories. (**b**) Conservation analysis of the mutated Glu in *dsr1*. (**c**) Amino acid sequence alignment of MDN1 homologues using WEBLOGO (http://weblogo.berkeley.edu/logo.cgi).

The results of the maximum-likelihood phylogenetic analysis of the amino acid sequences showed that MDN1 of Arabidopsis is homologous to those of *Camelina sativa, Capsella rubella, Eutrema salsugineum*, and *Brassica rapa* (Fig. 3a). Interestingly, these plants at the “d” sub-branch are all Brassicaceae. The “a” sub-branch species, containing *Pyrus* × *bretschneideri, Malus* × *domestica*, and *Fragaria vesca subsp. vesca*, are Rosaceae. Moreover, the “b, c, e, and f” sub-branches are Cucurbitaceae, Legumes, Solanaceae, and Poaceae, respectively (Fig. 3a). These observations suggest that MDN1 is conserved during plant evolution.

We further performed amino acid sequence alignment analysis of MDN1 homologues in plants, yeast, animals, and human. The results showed that the AAA and MIDAS domains were highly conserved, while the linker domain was weakly conserved (Supplementary Fig. 2a). However, the mutated amino acid at the 3838th position of AtMDN1 in *dsr1* (Glu) was extremely conserved among the detected species (Fig. 3b,c; Supplementary Fig. 2b). Considering the development defects of *dsr1*, we suggest that the Glu residue plays a role in maintaining AtMDN1 functions.

### Expression patterns of *AtMDN1*

**Figure 4:**
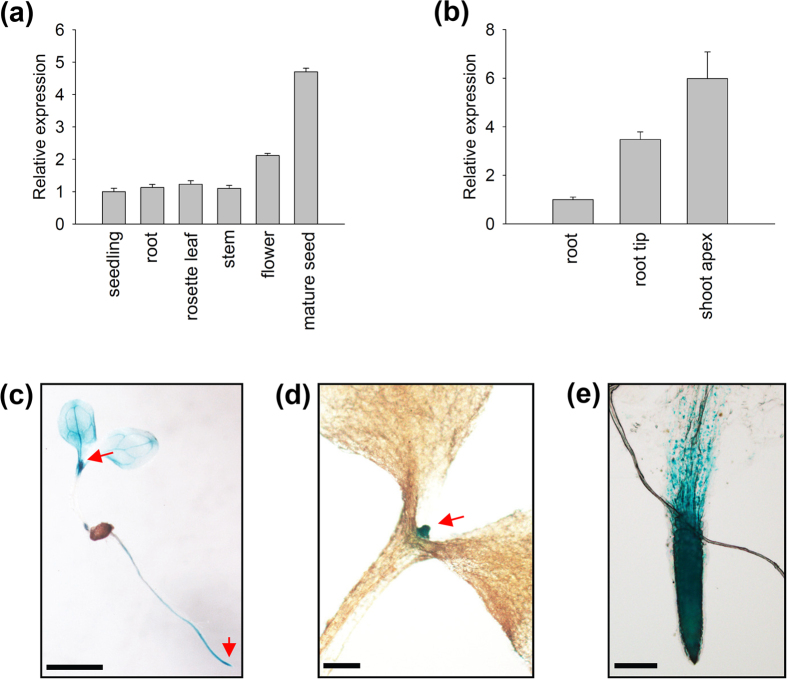
Expression pattern of *AtMDN1*. (**a**) Relative expression analysis of *AtMDN1* in the indicated tissues. Total RNA was isolated from 5-d-old seedlings, the stems of 40-d-old plants, the roots of 10-d-old plants, the rosette leaves of 20-d-old plants, the flowers of 60-d-old plants, and mature seeds (10 days after pollination). (**b**) Transcript abundance analysis of *AtMDN1* in the root tip and shoot apex. Total RNA was isolated from the roots, root tips, and shoot apices of 10-d-old plants. (**c**) GUS staining analysis of *AtMDN1* in a 5-d-old seedling. Bar = 5 mm. (**d**) GUS staining analysis of *AtMDN1* in the shoot apex of a 5-d-old seedling. Bar = 1 mm. (**e**) GUS staining analysis of *AtMDN1* in the root tip of a 5-d-old seedling. Bar = 100 μm.

The expression pattern of *AtMDN1* in plant tissues was initially determined using transcript analyses. The results showed that the *AtMDN1* transcripts could be clearly detected in various plant tissues, including root, stem, leaf, and so on (Fig. 4a). In mature seeds, the transcriptional levels of *AtMDN1* were significantly higher than those in other tissues. Notably, in tissues containing actively dividing cells, such as the shoot apex and root tip, the transcriptional levels of *AtMDN1* were obviously high (Fig. 4b). To further confirm these results, histochemical GUS reporter assays were performed. The results suggest that *AtMDN1* was expressed in the whole seedling, excluding the hypocotyl (Fig. 4c). In the shoot apex and root tip, GUS activity was substantially high (Fig. 4d,e).

We also investigated the expression pattern of *AtMDN1* using Genevestigator (a publicly available gene expression database, www.genevestigator.com). As shown in Supplementary Fig. 3, the RNA level of *AtMDN1* was easily detected in the shoot apex, seed embryo, stigma, root meristemoid zone, and endosperm. However, in the root elongation zone or pollen, the *AtMDN1* expression level was low. Based on these results, we concluded that *AtMDN1* is expressed in different tissues, particularly in the shoot apex and root tip, which are associated with the phenotypes of *dsr1*.

### AtMDN1 dysfunction affects various biological processes

**Figure 5:**
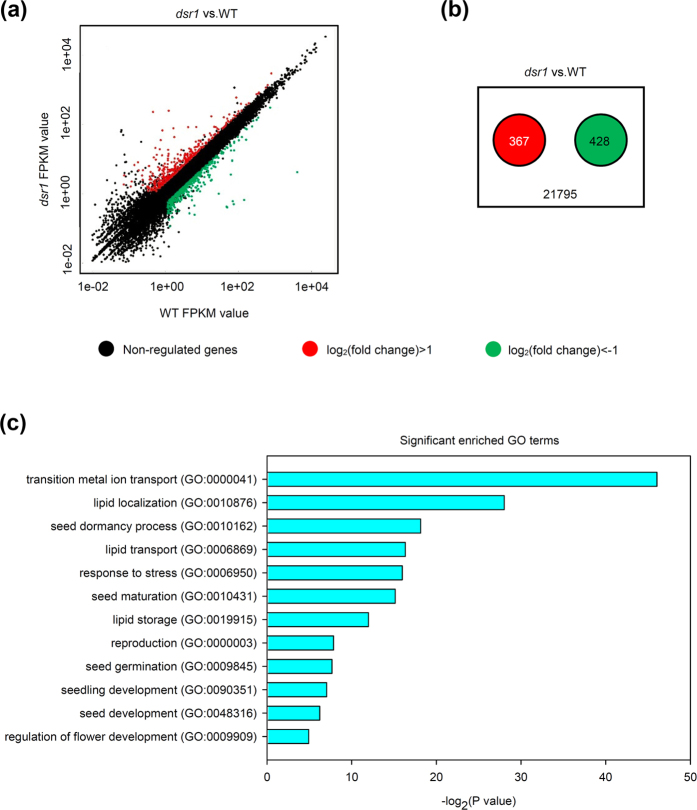
Comparison of the transcript profiles between wild type and *dsr1*. (**a**) Scatter plot of FPKM values for all detected genes in wild type and *dsr1* plants. The red plots indicate the significantly up-regulated genes, while the green plots indicate the down-regulated genes (*P* < 0.05). (**b**) The number of differentially expressed genes significantly up-regulated (red) or down-regulated (green) in the *dsr1* mutant. (**c**) GO terms significantly enriched (*P* < 0.05) with the differentially expressed genes in the *dsr1* mutant. The GO terms were sorted based on *P* values.

To further understand the mechanisms of *dsr1* development defects, we performed transcript profile analyses of 5-d-old *dsr1* and wild-type seedlings using RNA sequencing (RNA-seq). Three biological replicates were performed, and the results of Pearson’s correlation analysis among samples suggested that these data were reliable (Supplementary Fig. 4). The significantly differentially expressed genes were screened based on the fold-change (>2) of the fragments per kilobase per million fragments (FPKM) as well as the *P* values (<0.05, Fig. 5A). The results showed that among the detected 21795 gene transcripts, 367 genes showed significantly increased expression, while the expression of 428 genes was decreased in *dsr1* (Fig. 5b). Furthermore, the results of the Gene Ontology (GO) enrichment analysis of these differentially expressed genes indicated that the most significantly enriched GO term is GO: 0000041 (transition metal ion transport, Fig. 5c). Reflecting the MIDAS domain at the C-terminal end, these findings suggest that AtMDN1 plays potential roles in ion transport. Moreover, the dysfunction of AtMDN1 in *dsr1* also affects genes related to plant reproduction, seed development, maturation, dormancy, germination, and seedling development (Fig. 5c). These results were further confirmed by qRT-PCR analysis of randomly selected genes related to metal ion transport (GO: 0000041), seed dormancy process (GO: 0010162), plant reproduction (GO: 0000003), and seedling development (GO: 0090351, Fig. 6). The results indicated that AtMDN1 plays roles in various biological functions as well as the regulation of plant growth and development.

## Discussion

In organisms, AAA-type ATPase is involved in various functions, such as proteolytic activity, microtubule disassembly, membrane fusion, mitochondrial functions, pre-ribosome maturation, and peroxisome biosynthesis^16^,^17^. In the present study, we demonstrated that the developmental phenotypes of *dsr1* reflected a site mutation in AtMDN1, the largest AAA-ATPase in Arabidopsis (Fig. 2). In plants, the AAA-ATPase family plays critical roles in cellular activities and physiological functions. For instance, the homozygote of *atskd1* is lethal, reflecting the fact that AtSKD1, an AAA-ATPase localized to multivesicular bodies, plays a role in the maintenance of the large central vacuole of plant cells^18^. RPT2a, as an AAA-ATPase associated with the 26S proteasome, activates both morphological and defence signals through interactions with UNI/uni1-D^19^. The loss of RPT2a results in a weak defect in 26S proteasome activity and the enlargement of plant organs^20^. A recent study also reported that transgenic lines overexpressing the mitochondrial outer membrane AAA-ATPase, AtOM66, exhibit strong leaf curling, reduced starch content, and increased salicylic acid content phenotypes^21^. Moreover, in a previous study, we showed that a homologue of AtMDN1 in Arabidopsis, AtSAG, plays roles in seed germination and seedling development through the negative regulation of ABA signalling^22^. Consequently, AAA-ATPase activity is responsible for plant growth and development.

The *dsr1* mutant was screened from a mutant library built by our group. The results of map-based cloning showed that the mutation site is the 3838th amino acid, Glu, located in the linker domain of AtMDN1 (Fig. 2b). Intriguingly, two additional amino acids right next to the Glu, Gly and Phe, are also highly conserved in the detected species, suggesting a key ‘GEF’ motif (Fig. 3b,c; Supplementary Fig. 2b). In most organisms, the AAA and MIDAS domains are highly conserved (Supplementary Fig. 2a), but the sequence of the linker domain ranges between 1700 and 2300 residues, is weakly conserved, and predicted to be essential for protein structure diversity^10^. In most plants, MDN1 is translated from one cDNA sequence. However, in rice, the OsMDN1 protein is divided into two parts. One part, containing the AAA and linker domains, is derived from *Os10g31864*, while the other part, containing the MIDAS domain, is derived from *Os10g31856*. Interestingly, the ‘GEF’ motif is also present in the linker domain of OsMDN1. Although there are no studies on the functions of OsMDN1, we propose that these two parts might interact to fulfil protein functions *in vivo*. Thus, we speculated that the ‘GEF’ motif might play critical roles in the maintenance of MDN1 function. However, there is one exception. In apple, part of the linker domain containing the GEF motif was not detected (Fig. 3b), which might reflect a deletion of the MIDAS domain in MdMDN1.

**Figure 6:**
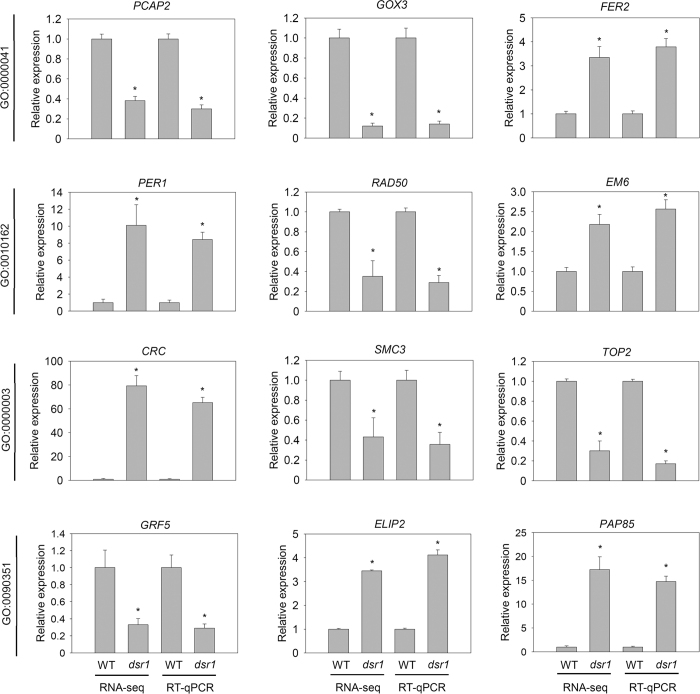
qRT-PCR analysis of randomly selected differentially expressed genes. qRT-PCR analysis of the genes related to metal ion transport (GO: 0000041; *PCAP2, GOX3*, and *FER2*), seed dormancy (GO: 0010162; *PER1, RAD50*, and *EM6*), plant reproduction (GO: 0000003; *CRC, SMC3*, and *TOP2*), and seedling development (GO: 0090351; *GRF5, ELIP2*, and *PAP85*) in wild type and *dsr1* plants. Total RNA was isolated from 5-d-old seedlings grown under normal growth conditions. *Actin2* was used as an internal control. The data are presented as the mean values of three biology repeats, and the error bars indicate the SD. Significant differences were calculated using Student’s *t*-test (^*^P < 0.01).

*AtMDN1* is highly expressed in tissues, such as the shoot apex and root tip (Fig. 4), suggesting its potential roles in cell division. Under normal growth conditions, both the shoots and roots of *dsr1* exhibit dwarf phenotypes (Fig. 1). The maize mutant dek* shows similar phenotypes, reflecting a delay in plant development according to decreased ZmRea1 activity^15^. However, the phenotype of *dsr1* does not merely reflect delayed development, as the roots of 3-week-old plants were also severely short (Fig. 1d). In dek* endosperm, the transcription of ribosome biogenesis, translational elongation, and nucleosome-related genes was significantly affected. However, in *dsr1* seedlings, the above three GO terms were not significantly enriched with differentially expressed genes (GO: 0005840, ribosome, *P *= 0.98; GO: 0006414, translational elongation, *P *= 0.19; GO:0000786, nucleosome, *P *= 0.43), while the expression of genes related to metal ion transport and plant development was severely impaired (Fig. 5c). In dek*, the mutation site of ZmRea1 (ZmMDN1) is located at the region between the AAA-ATPase and linker domain. Thus, on the one hand, the dramatic differences in the GO enrichment of differentially expressed genes might reflect the different tissues used for RNA isolation. On the other hand, the different mutation sites in MDN1 of dek* and *dsr1* might also induce differences in the transcript profiles through the altered functions of different protein domains. According to the RNA-seq results, the dysfunction of AtMDN1 in *dsr1* affects the transcription of many genes related to plant growth and development (Figs 5 and 6). These cellular responses might be directly or indirectly triggered by ribosome signals because of the potential functions of AtMDN1 in pre-ribosome maturation, which require further examination.

In the present study, we clarified that *dsr1* phenotypes reflect a Glu mutation in the linker domain of AtMDN1. However, the mechanism of the Glu or the GEF motif in maintaining protein function remains unknown. Nevertheless, based on previous studies of the ribosome in yeast, we hypothesized that some sites, such as the GEF motif, might be essential for the flexibility of the linker domain, which plays a role in MIDAS domain interactions with other pre-ribosome proteins, such as NLE (a homologue of Rsa4)^23^. These results suggest that MDN1 is tightly associated with plant growth and production. Thus, defining the protein structure and function of MDN1 will be useful for crop research.

## Methods

### Plant Materials and Growth Conditions

The wild-type plant used in the present study is *Arabidopsis thaliana* Col-0. The heterozygous mutant SALK_057010 was obtained from the ABRC stock. NaClO (0.1%) and ethanol (70%) were used for seed sterilization. The plants were grown on solid 1/2 Murashige and Skoog (MS) medium (pH 5.7) or in soil, with 16 h light/8 h dark cycles and 100 μM m^−2^ s^−1^ photon flux density at 22~24 °C.

### Plant Phenotype Analyses

For germination rate analysis, surface sterilized seeds were plated on 1/2 MS medium as described above. The plates were stratified at 4 °C in darkness for 3 days and subsequently transferred to normal growth conditions as described above. The number of plants with open green cotyledons was recorded to calculate the germination rate at days 2–5. Six independent experiments were performed, and each experiment contained five replications. For plant height determination, 5-week-old plants grown under normal growth conditions were collected and the primary stem lengths were measured. To observe the seed set, mature siliques were cleared with 90% ethanol for at least 2 h (Fig. 1h right) or not (Fig. 1h left) and subsequently photographed using a stereomicroscope (SZX7, OLYMPUS).

### Sequence Analysis

The protein sequence of AtMDN1 was employed to search for homologues in the available sequence database (http://blast.ncbi.nlm.nih.gov/). Multiple sequence alignment was performed using ClustalX (Plate-Forme de Bio-Informatique, Illkirch Cedex, France) and DNAMAN 8.0. Phylogenetic analysis was performed using MEGA 5 (Statistical method: Maximum Likelihood; Model: Jones-Taylor-Thornton model).

### GUS staining analysis

A plasmid containing the AtMDN1 promoter was constructed after subcloning a 2.0-kb fragment upstream of the translation start site into the pBI121 vector. Subsequently, the construct was introduced into *Agrobacterium tumefaciens* GV3101 for plant transformation. The transgenic plants were selected on 1/2 MS medium containing 50 mg L^−1^ kanamycin. The tissues of transgenic plants were collected and immersed in 90% acetone for 20 min at 4 °C. The tissues were washed twice with GUS elution buffer (0.1 M PBS, 10 mM EDTA, 2 mM potassium ferrocyanide, 2 potassium ferricyanide, and 0.1% Triton X-100, pH 7.0) and subsequently immersed in GUS staining buffer (1 mg x-Gluc in 1 mL elution buffer). The tissue samples were stained for 48 h at 37 °C. The tissues were cleaned and destained in ethanol (70%) and subsequently stored at 4 °C until further examination. Six independent transgenic lines were tested, and the typical results are shown.

### Real-time quantitative RCR

RNA extraction and cDNA synthesis experiments were performed as described previously^24^. The qRT-PCR experiments were performed using a CFX96 real-time PCR system (Bio-Rad, C1000) with SYBR green real-time PCR master mix (Takara). The detection of each gene transcript was performed for at least three biology replicates, and each bio-replicate contained three technical replicates. All of the primers are listed in Supplementary Table 1.

### RNA-sequencing

Whole 5-d-old seedlings were sampled for the RNA-seq experiments. High-throughput sequencing was performed at CapitalBio Technology (Beijing, China). The data were extracted according to a standard protocol. The differentially expressed genes (up- or down-regulated) were screened according to the log-fold changes (*dsr1* vs. WT) and subsequently analysed using the Arabidopsis Information Resource Database (http://www.Arabidopsis.org) and Gene Ontology Database (http://www.geneontology.org). The results of RNA-seq were confirmed using qRT-PCR experiments. GO enrichment analysis was performed using online software (http://kobas.cbi.pku.edu.cn/), and significance analysis was performed according to the equation of the hypergeometric distribution: 
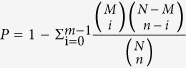
, where *N* is the total number of genes annotated by GO in these samples; *n* is the number of differentially expressed genes annotated by GO; *M* is the number of genes annotated by some specific GO term in all of the detected genes; and *m* is the number of differentially expressed genes annotated by some specific GO term.

### Accession Numbers

Sequence data of this article can be found in the *Arabidopsis* Genome Initiative or GenBank/EMBL data libraries under the following accession numbers: *MDN1*, AT1G67120; *PCAP2*, AT5G44610; *GOX3*, AT4G18360; *FER2*, AT3G11050; *PER1*, AT1G48130; *RAD50*, AT2G31970; *EM6*, AT2G40170; *CRC*, AT1G69180; *SMC3*, AT2G27170; *TOP2*, AT5G10540; *GRF5*, AT3G13960; *ELIP2*, AT4G14690; *PAP85*, AT3G22640.

## Additional Information

**How to cite this article**: Li, P.-C. *et al*. The Mutation of Glu at Amino Acid 3838 of AtMDN1 Provokes Pleiotropic Developmental Phenotypes in Arabidopsis. *Sci. Rep.* **6**, 36446; doi: 10.1038/srep36446 (2016).

**Publisher’s note:** Springer Nature remains neutral with regard to jurisdictional claims in published maps and institutional affiliations.

## Supplementary Material

Supplementary Information

## References

[b1] Ulbrich C. . Mechanochemical removal of ribosome biogenesis factors from nascent 60S ribosomal subunits. Cell 138, 911–922 (2009). 19737519 10.1016/j.cell.2009.06.045

[b2] Kressler D., Hurt E., Bergler H. & Bassler J. The power of AAA-ATPases on the road of pre-60S ribosome maturation--molecular machines that strip pre-ribosomal particles. Biochim. Biophys. Acta. 1823, 92–100 (2012). 21763358 10.1016/j.bbamcr.2011.06.017 PMC3264779

[b3] Matsuo Y. . Coupled GTPase and remodelling ATPase activities form a checkpoint for ribosome export. Nature 505, 112–116 (2014). 24240281 10.1038/nature12731 PMC3880858

[b4] Baßler J. . A network of assembly factors is involved in remodeling rRNA elements during preribosome maturation. J. Cell Biol. 207, 481–498 (2014). 25404745 10.1083/jcb.201408111 PMC4242840

[b5] Strunk B. S. & Karbstein K. Powering through ribosome assembly. RNA 15, 2083–2104 (2009). 19850913 10.1261/rna.1792109 PMC2779681

[b6] Staley J. P. & Woolford J. L. Assembly of ribosomes and spliceosomes: complex ribonucleoprotein machines. Curr. Opin. Cell Biol. 21, 109–118 (2009). 19167202 10.1016/j.ceb.2009.01.003 PMC2698946

[b7] Granneman S. & Baserga S. J. Ribosome biogenesis: of knobs and RNA processing. Exp. Cell Res. 296, 43–50 (2004). 15120992 10.1016/j.yexcr.2004.03.016

[b8] Nissan T. A. . A pre-ribosome with a tadpole-like structure functions in ATP-dependent maturation of 60S subunits. Mol. cell 15, 295–301 (2004). 15260980 10.1016/j.molcel.2004.06.033

[b9] Baßler J. . The AAA-ATPase Rea1 drives removal of biogenesis factors during multiple stages of 60S ribosome assembly. Mol. cell 38, 712–721 (2010). 20542003 10.1016/j.molcel.2010.05.024 PMC3372891

[b10] Garbarino J. E. & Gibbons I. Expression and genomic analysis of midasin, a novel and highly conserved AAA protein distantly related to dynein. BMC Genomics 3, 18 (2002). 12102729 10.1186/1471-2164-3-18 PMC117441

[b11] Galani K., Nissan T. A., Petfalski E., Tollervey D. & Hurt E. Rea1, a dynein-related nuclear AAA-ATPase, is involved in late rRNA processing and nuclear export of 60S subunits. J. Biol. Chem. 279, 55411–55418 (2004). 15528184 10.1074/jbc.M406876200

[b12] Baßler J. . Identification of a 60S preribosomal particle that is closely linked to nuclear export. Mol. cell 8, 517–529 (2001). 11583615 10.1016/s1097-2765(01)00342-2

[b13] Barrio-Garcia C. . Architecture of the Rix1-Rea1 checkpoint machinery during pre-60S-ribosome remodeling. Nat. Struct. Mol. Biol. 23, 37–44 (2016). 26619264 10.1038/nsmb.3132

[b14] Chantha S.-C., Gray-Mitsumune M., Houde J. & Matton D. P. The *MIDASIN* and *NOTCHLESS* genes are essential for female gametophyte development in Arabidopsis thaliana. Physiol. Mol. Biol. Plants 16, 3–18 (2010). 23572950 10.1007/s12298-010-0005-y PMC3550630

[b15] Qi W. . Maize reas1 mutant stimulates ribosome use efficiency and triggers distinct transcriptional and translational responses. Plant Physiol. 170, 971–988 (2016). 26645456 10.1104/pp.15.01722 PMC4734584

[b16] Joly N., Zhang N. & Buck M. ATPase site architecture is required for self-assembly and remodeling activity of a hexameric AAA+ transcriptional activator. Mol. cell 47, 484–490 (2012). 22789710 10.1016/j.molcel.2012.06.012 PMC3419264

[b17] Neuwald A. F., Aravind L., Spouge J. L. & Koonin E. V. AAA+: A class of chaperone-like ATPases associated with the assembly, operation, and disassembly of protein complexes. Genome Res. 9, 27–43 (1999). 9927482

[b18] Shahriari M. . The AAA-type ATPase AtSKD1 contributes to vacuolar maintenance of Arabidopsis thaliana. Plant J. 64, 71–85 (2010). 20663085 10.1111/j.1365-313X.2010.04310.x

[b19] Chung K. & Tasaka M. RPT2a, a 26S proteasome AAA-ATPase, is directly involved in Arabidopsis CC-NBS-LRR protein uni-1D-induced signaling pathways. Plant Cell Physiol. 52, 1657–1664 (2011). 21791544 10.1093/pcp/pcr099

[b20] Kurepa J. . Loss of 26S proteasome function leads to increased cell size and decreased cell number in Arabidopsis shoot organs. Plant Physiol. 150, 178–189 (2009). 19321709 10.1104/pp.109.135970 PMC2675745

[b21] Zhang B. . The mitochondrial outer membrane AAA ATPase AtOM66 affects cell death and pathogen resistance in Arabidopsis thaliana. Plant J. 80, 709–727 (2014). 25227923 10.1111/tpj.12665

[b22] Chen C. . Arabidopsis SAG protein containing the MDN1 domain participates in seed germination and seedling development by negatively regulating *ABI3* and *ABI5*. J. Exp. Bot. 65, 35–45 (2014). 24163287 10.1093/jxb/ert343 PMC3883281

[b23] Chantha S.-C. & Matton D. P. Underexpression of the plant *NOTCHLESS* gene, encoding a WD-repeat protein, causes pleitropic phenotype during plant development. Planta 225, 1107–1120 (2007). 17086402 10.1007/s00425-006-0420-z

[b24] Li P.-C. . Arabidopsis YL1/BPG2 is involved in seedling shoot response to salt stress through ABI4. Sci. Rep. 6, 30163 (2016). 27444988 10.1038/srep30163 PMC4957093

