# Supplementary material for: The Mutation of Glu at Amino Acid 3838 of AtMDN1 Provokes Pleiotropic Developmental Phenotypes in Arabidopsis
Source: Sci Rep. 2016 Nov 8;6:36446. doi: 10.1038/srep36446 (PMC5099923; doi:10.1038/srep36446)
Supplement: Supplementary Information [file srep36446-s1.pdf]

Title: The Mutation of Glu at Amino Acid 3838 of AtMDN1 Provokes Pleiotropic Development Phenotypes in Arabidopsis

Peng-Cheng Li[1,2†], Shao-Wei Yu[1†], Ke Li[3], Jin-Guang Huang[1], Xing-Jun Wang[2], and Cheng-Chao Zheng[1]*

Affiliations:

[1]State Key Laboratory of Crop Biology, College of Life Sciences, Shandong Agricultural University, Tai'an, Shandong, PR China

[2]Bio-Tech Research Center, Shandong Academy of Agricultural Sciences, Shandong Provincial Key Laboratory of Crop Genetic Improvement, Ecology and Physiology, Jinan, Shandong, PR China

[3]College of Life Sciences, Shandong University，Ji'nan, Shandong, PR China

[†]These authors contributed equally to this work.

*Correspondence and requests for materials should be addressed to C-C.Z. (e-mail: cczheng@sdau.edu.cn)

Supplementary information

Supplementary Figure 1. Phenotype of *mdn1* heterozygote.

Supplementary Figure 2. Conservation analysis of MDN1 proteins.

Supplementary Figure 3. Expression pattern analysis of *AtMDN1* using public database.

Supplementary Figure 4. Pearson correlation analysis of different samples. rep, repeats.

Supplementary Table 1. Key primers used in this study.

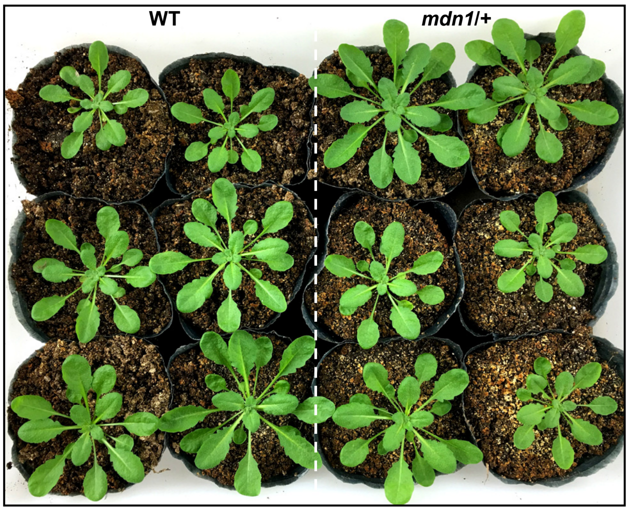

Supplementary Figure 1. Phenotype of *mdn1* heterozygote.

Wild type and heterozygote of *mdn1* are grown for 4 weeks under normal conditions.

a

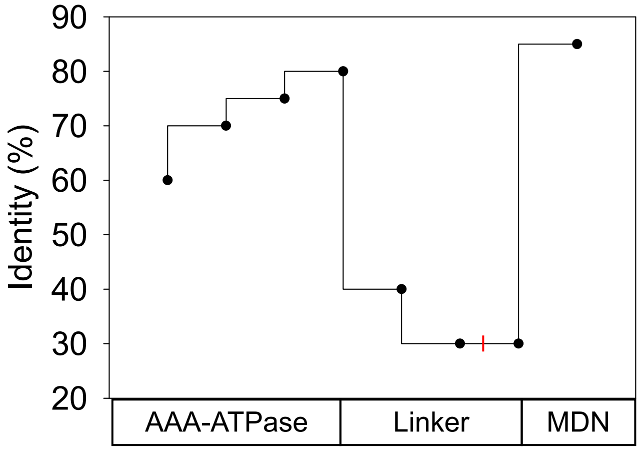

b

```
A. thaliana    ISQSLVEFIETSNVGEFRRRLQLLFCFLLQL
D. rerio       VSSSLQAFIEGSTLGEFSVRLSMLLGFHCHI
S. cerevisiae  LVSSLNLFFSKSTLGEFNARLDLVKAFYKHI
M. musculus    LVSTLQAFIEGSSLGEFHVRLQMLLVFHCHV
H. sapiens     LVSTLQAFIEGSSLGEFHVRLQMLLVFHCHV
                             GEF
```

Supplementary Figure 2. Conservation analysis of MDN1 proteins.

(**a**) Conservation analysis of the AAA, the linker, and the MIDAS (MDN) domains in MDN1 homologues of organisms showed in **b** and Fig. 3**a**. The red bar indicates the mutation site of *dsr1*.

(**b**) Conservation analysis of the GEF motif in MDN1 homologues of *Danio rerio* (XP_003200751.2), *Saccharomyces cerevisiae* (NP_013207.1), *Mus musculus* (XP_006537539.1) and *Homo sapiens* (NP_055426.1).

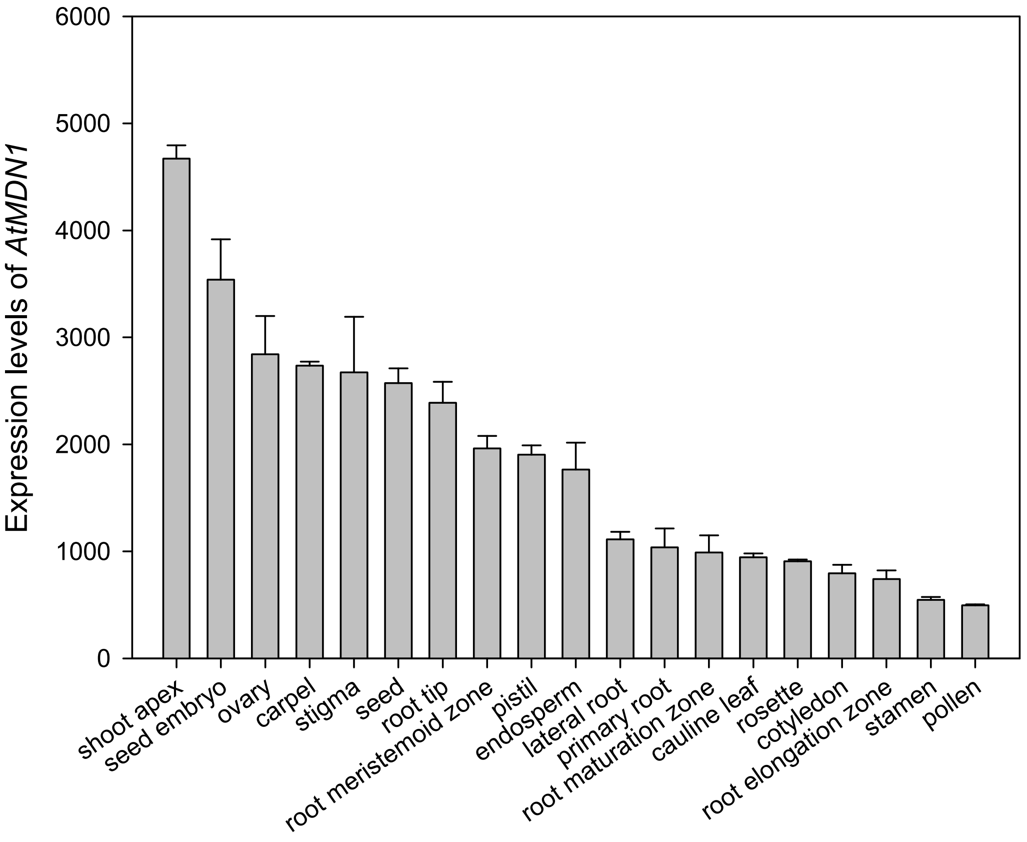

Supplementary Figure 3. Expression pattern analysis of *AtMDN1* using public database.

Tissue-specific expression pattern of *AtMDN1*. The data are collected from Genevestigator.

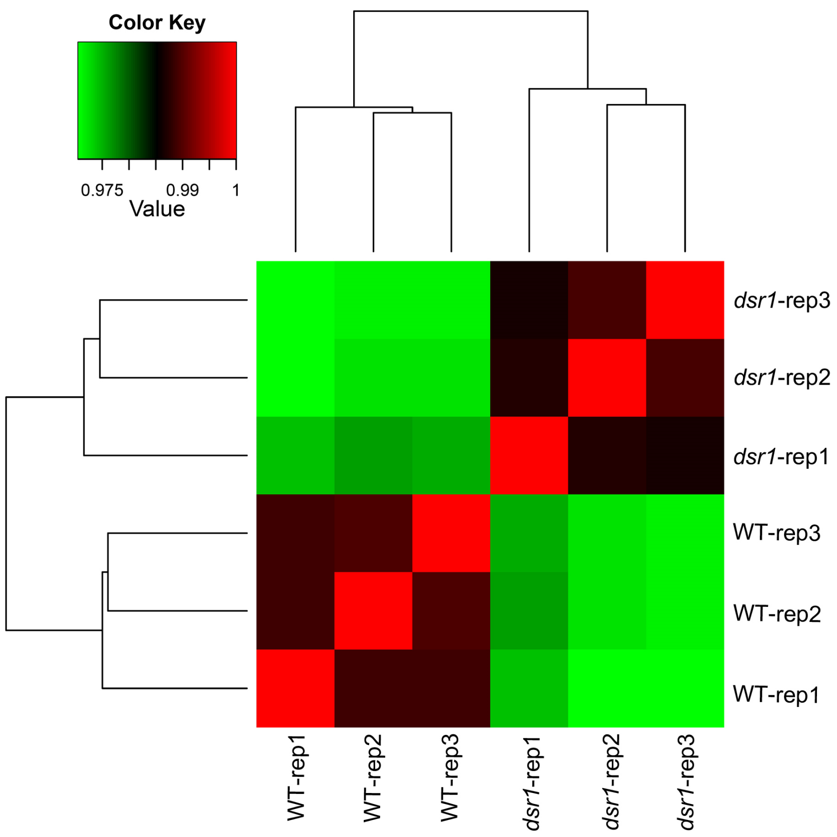

Supplementary Figure 4. Pearson correlation analysis of different samples. rep, repeats.

Supplementary Table 1. Key primers used in this study.

Purpose	Primer name	Sequence (5'-3')
AtMDN1 promoter	forward (P1)	ATCCAAAATGGTCAGCTG
	reverse (P2)	TGAAACCTGTTGACAAAC
qRT- PCR	*MDN1*-forward	GTCACATGATCGGTCTAG
	MDN1-reverse	CCCTGTTTCTCCTACTAAG
	PCAP2-forward	ACCGGAGGAAATAATTGC
	PCAP2-reverse	CTGGCTTCTTCTCTTCCA
	GOX3-forward	GATGGAATATGAGAAGATCG
	GOX3-reverse	GGCATAGAAATATTAAACCCTA
	FER2- forward	CTACCCCTTTTGTTTCTC
	FER2- reverse	CTGTCAAAGTAGGCATAC
	PER1- forward	GGTCCTGACAGTAAGATAA
	PER1- reverse	GTGGCGATCTTGTTATTG
	RAD50- forward	GACACTGAATCGAGAAAG
	RAD50- reverse	CCTGTTCCGTCTTCTTTA
	EM6-forward	GAAGGATATCAGCAGATG
	EM6-reverse	GGTCCTGAATTTGGATTC
	CRC-forward	TGGTGGAAGTGACTATAAGA
	CRC-reverse	TCTGCTTCTTCTCAGGAG
	SMC3-forward	CTCGTGCTAATCCTTACTA
	SMC3-reverse	TCCGTTTATTTCCTGTTTC
	TOP2-forward	GACCTTACTTCTCATTGC
	TOP2-reverse	GCACAACATCAATTCCAA
	GRF5-forward	CTCTCATTCACCAACAAC
	GRF5-reverse	AGCAGAATAAGTAGTAGAAGA
	ELIP2-forward	CAAGCCTAAGGTGAGTAC
	ELIP2-reverse	TCCCTTTGATAACTCCATG
	PAP85-forward	TCTCCTTATCTCCATAGC
	PAP85-reverse	GGAACCAATCACTGAAAC
	Actin2-forward	CTTCGTCTTCCACTTCAG
	Actin2-reverse	ATCATACCAGTCTCAACAC
